# On the validity of *Plecturocebus parecis* Gusmão et al., 2019, a titi monkey described from southwestern Brazilian Amazonia

**DOI:** 10.1007/s10329-026-01288-7

**Published:** 2026-09-18

**Authors:** Almério Câmara Gusmão, Odair Diogo da Silva, Gerson Buss, Leandro Jerusalinsky, Renata Bocorny de Azevedo, Stephen F. Ferrari, Manoel dos Santos Filho, Adrian A. Barnett

**Affiliations:** 1 Instituto Federal de Educação, Ciência e Tecnologia de Rondônia (IFRO), Campus Cacoal, RO Brazil; 2 Graduate Program in Environmental Sciences, Universidade do Estado de Mato Grosso (UNEMAT), Cáceres, MT Brazil; 3 Instituto Chico Mendes de Conservação da Biodiversidade (ICMBio), Centro Nacional de Pesquisa e Conservação de Primatas Brasileiros (CPB), Cabedelo, PB Brazil; 4 Department of Ecology, Universidade Federal de Sergipe (UFS), São Cristóvão, SE Brazil; 5 Instituto Nacional de Pesquisas da Amazônia (INPA), Manaus, AM Brazil

**Keywords:** Callicebinae, Hybridization, Species delimitation, Geographic variation, Amazonia

## Abstract

*Plecturocebus parecis* was described from the southwestern Brazilian Amazon, but its taxonomic status was subsequently questioned after mitochondrial evidence prompted alternative hypotheses regarding species limits within this complex. We reassessed these hypotheses using 57 occurrence records obtained from 47 localities during 18 field expeditions conducted between 2023 and 2025. Morphologically homogeneous populations exhibiting the diagnostic phenotype of *P. parecis* occupied a broad geographic area centred on the Juína region, whereas intermediate phenotypes were geographically restricted to peripheral portions of the distribution, particularly within the Comemoração and Roosevelt drainages. This spatially structured pattern of phenotypic variation provides stronger support for the interpretation of *P. parecis* as a geographically cohesive and diagnosable lineage than for hypotheses proposing a broadly hybrid population lacking taxonomic cohesion. The distributions of *P. parecis*, *P. cinerascens*, and *P. bernhardi* were also geographically structured, with only limited areas of potential contact. Although additional nuclear genomic and cytogenetic data remain necessary to distinguish among alternative evolutionary scenarios, our results provide a stronger empirical basis for evaluating the recognition of *P. parecis* while highlighting the importance of comprehensive geographic sampling when evaluating species boundaries in Amazonian primates.

## Introduction

Species delimitation remains a central challenge in primate taxonomy, particularly in regions characterized by recent diversification, incomplete lineage sorting, and secondary contact. In Amazonian primates, closely related taxa often exhibit parapatric distributions, narrow zones of sympatry, and localized hybridization, making species boundaries difficult to interpret when based solely on single lines of evidence. These challenges have historically fostered different taxonomic approaches, often framed as “splitting” and “lumping” strategies. While lumping approaches emphasize broad population sampling and genetic continuity, splitting approaches emphasize diagnosability, geographic structure, and the recognition of recently diverged lineages as testable species-level hypotheses. Both perspectives are widely used in primate systematics, particularly in regions of high spatial and ecological heterogeneity.

In recently diverged taxa, hybridization and morphological intermediacy may occur even when populations otherwise maintain ecological or geographic cohesion, and remain diagnosable across all other areas of their distribution. Integrative approaches that combine morphology, geography, and ecology have therefore been increasingly applied in primate systematics and conservation assessments, particularly in biodiversity hotspots undergoing rapid environmental change.

Within this context, the taxonomic status of *Plecturocebus parecis* Gusmão et al. 2019 has been debated. The species was originally described based on morphological and geographic evidence, complemented by genetic analyses conducted under a Bayesian framework that documented partial genetic similarity with neighboring taxa without rejecting the diagnosability of the lineage (Gusmão et al. 2019). Subsequent studies proposed alternative interpretations to explain the discordance between mitochondrial, morphological, and geographic evidence in *Plecturocebus parecis*, highlighting the need for broader geographic and morphological data to evaluate species limits within this complex (Byrne et al. 2021). Here, we present an expanded geographic and morphological dataset to characterize patterns of phenotypic variation across the distribution of *P. parecis* and provide new empirical evidence relevant to species delimitation in this taxonomic complex.

## Methods

We conducted fieldwork aimed at documenting *Plecturocebus parecis* and related species during one expedition in 2023 (February) and seventeen expeditions in 2025 (February–October). We considered expeditions as discrete field campaigns involving travel to a focal region, during which we conducted multiple standardized surveys, systematically searching for and recording individuals along roads, trails, and forest edges within selected localities.

**Fig. 1:**
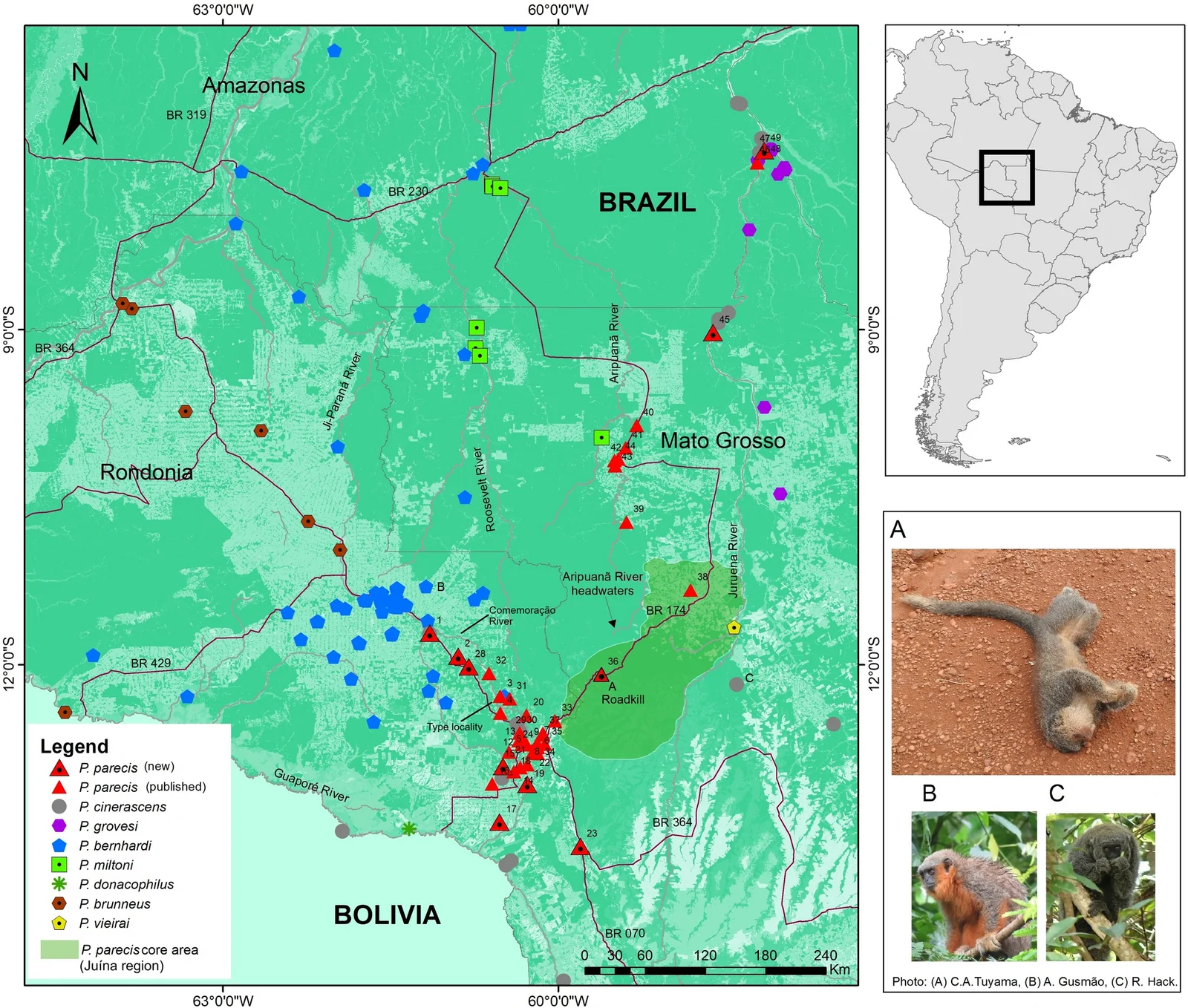
Distribution of *Plecturocebus* in southwestern Amazonia based on published records (Gusmão et al. 2019; Byrne et al. 2021) and 57 new field observations collected during 18 expeditions (2023–2025). Symbols indicate records of *P. parecis* (*n* = 41), *P. cinerascens* (*n* = 10), and *P. bernhardi* (*n* = 6). Insets show representative individuals: (**A**) typical *P. parecis* from the Juína region, exhibiting the diagnostic greyish-white sideburns and anterior thorax contrasting with a reddish-brown dorsum; (**B**) typical *P. bernhardi*, characterized by continuous orange coloration across the chest and limbs; (C) typical *P. cinerascens*, showing uniformly grey pelage

Survey sites spanned ecological transitional zones from the type locality of *Plecturocebus parecis* in Pimenta Bueno municipality (Rondônia state, Brazil) to the slopes of the Parecis Highlands, including the headwaters of the Comemoração and Apidiá rivers, tributaries of the Ji-Paraná/Machado and Roosevelt rivers. Total sampling effort comprised approximately 150 km of survey transect across 47 localities, including the mouth of the Juruena River within Juruena National Park (Fig. 1).

We based our assessment of morphological variation on criteria previously proposed in the literature (Auricchio 2010). These assessments were based on in-field photographic records, direct examination of a *Plecturocebus parecis* specimen found road-killed along BR-174 in an area occupied by morphologically homogeneous populations of the species, and comparative analyses of two specimens housed in the Universidade do Estado de Mato Grosso Mammalogy Collection (*P. cinerascens*, UNEMAT-ODS 009; *P. bernhardi*, UNEMAT-MSF 2687). We identified species using visual diagnosis of pelage characters, high-resolution photographs, and comparisons with published species descriptions (Gusmão et al. 2019).

## Results

We obtained 57 records of *Plecturocebus* (*Plecturocebus parecis*, 41; *P. cinerascens*, 10; *P. bernhardi*, 6) (Fig. 1). Across the 41 records attributed to *P. parecis*, central populations consistently exhibited a homogeneous pelage pattern, allowing clear distinction from adjacent taxa. All individuals recorded in these central areas shared the same combination of diagnostic traits, including greyish-white sideburns, forelimbs, and anterior thorax, contrasting with a reddish-brown dorsum and darker distal limbs.

Unlike the pelage pattern described above for *Plecturocebus parecis*, *P. cinerascens* (UNEMAT-ODS 009) displayed a uniformly gray pelage, whereas *P. bernhardi* (UNEMAT-MSF 2687) showed continuous orange coloration across the chest, limbs, and flanks. A road-killed specimen of *P. parecis* collected along BR-174 (Fig. 1) confirmed the presence of morphologically typical *P. parecis* near the Aripuanã River headwaters, where populations consistently exhibited the diagnostic phenotypic traits attributed to the species.

Our records reveal a more complex distributional pattern than previously documented (Fig. 1). Within the Comemoração and Roosevelt drainages and in the northernmost portion of the range of *Plecturocebus parecis*, we recorded individuals with contrasting pelage patterns within the same drainage systems, including fully dark individuals, typical white-bearded individuals, and others with reduced beard contrast. These heterogeneous phenotypes were geographically restricted and did not extend throughout the broader distribution of populations exhibiting the diagnostic phenotype of *P. parecis*. In contrast, central populations (Juína–Terra Roxa) were consistently morphologically cohesive across broad areas, showing little observable variation in diagnostic pelage traits.

## Discussion

Our expanded geographic and morphological sampling substantially expands the empirical basis available for evaluating species limits in *Plecturocebus parecis*. The resulting dataset reveals spatial patterns of phenotypic variation that were not apparent from previous sampling and provides an opportunity to re-evaluate competing hypotheses regarding species limits within this system (Byrne et al. 2021).

The available molecular, morphological, and geographic evidence has been interpreted as supporting multiple alternative hypotheses regarding the taxonomic status and evolutionary history of *Plecturocebus parecis* (Byrne et al. 2021). Rather than supporting a single explanation, these hypotheses reflect different interpretations of the observed phylogenetic, morphological, and distributional patterns, including scenarios involving geographic variation, hybridization, and alternative evolutionary histories. Distinguishing among these competing hypotheses therefore requires evaluating whether independent lines of evidence are more consistent with one interpretation than with the others. In this context, the geographically structured patterns of phenotypic variation documented here provide an independent basis for reassessing these alternative interpretations.

Phenotypic variation was not uniformly distributed across the study region. Instead, heterogeneous phenotypes were geographically restricted to peripheral portions of the distribution, particularly within the Comemoração and Roosevelt drainages, whereas populations from the central portion of the range consistently exhibited the diagnostic pelage characters originally attributed to *Plecturocebus parecis*. Central populations remained remarkably homogeneous across multiple localities despite the broad geographic extent sampled, whereas peripheral populations included typical white-bearded individuals, darker morphotypes, and intermediate phenotypes. This geographically structured distribution of phenotypic variation was not apparent from previous sampling and forms the basis for evaluating the alternative taxonomic hypotheses discussed below.

The alternative hypotheses proposed to explain the discordance between mitochondrial and morphological evidence in *Plecturocebus parecis* provide an important framework for interpreting the geographic patterns documented here (Byrne et al. 2021). Our expanded sampling indicates that the type locality, where this discordance was documented, lies near the northwestern limit of the broader distribution of morphologically homogeneous populations exhibiting the diagnostic phenotype of *P. parecis* and in geographic proximity to records of both *P. bernhardi* and *P. cinerascens*. This broader geographic context suggests that the evolutionary processes inferred from the type locality may not be representative of morphologically diagnosable populations throughout the species’ distribution. Consequently, our results strengthen the relative support for hypotheses recognizing *P. parecis* as a geographically cohesive and diagnosable lineage, while indicating that the localized discordance documented at the type locality should not be automatically generalized to the species as a whole.

More broadly, this case highlights the importance of comprehensive geographic sampling when evaluating competing species-delimitation hypotheses. As emphasized in recent discussions of species delimitation, distinguishing geographically structured intraspecific variation from independently evolving lineages depends on adequately characterizing the spatial distribution of phenotypic and genetic diversity (Burbrink et al. 2022). In this context, geographically restricted zones of phenotypic heterogeneity should be interpreted in light of the broader spatial organization of diagnostic phenotypes across the distribution, rather than as isolated evidence against taxonomic cohesion. Our results reinforce the importance of integrating morphology, geography, and molecular evidence when interpreting discordant evolutionary signals, particularly in recently diversified Amazonian primates.

Although additional genomic and cytogenetic data will be important for distinguishing among the evolutionary mechanisms underlying the mitochondrial discordance documented at the type locality, our expanded geographic and morphological dataset substantially strengthens the empirical basis for evaluating species limits in *Plecturocebus parecis*. By documenting the broader spatial distribution of morphologically homogeneous populations exhibiting the diagnostic phenotype of the species, our study provides a more robust geographic framework for interpreting discordant molecular signals. This integrative perspective is consistent with contemporary approaches to primate taxonomy that emphasize diagnosability, geographic structure, and multiple complementary lines of evidence when evaluating species limits (Vermeer et al. 2025).

## Conclusion

Our expanded field dataset shows that populations exhibiting the diagnostic phenotype of *Plecturocebus parecis* remain morphologically cohesive across a broad geographic portion of the species’ distribution, whereas phenotypic heterogeneity is confined to geographically restricted peripheral areas. This broader geographic perspective provides stronger support for interpreting *P. parecis* as a geographically cohesive and diagnosable lineage while indicating that evolutionary inferences based primarily on the type locality should be interpreted within the broader geographic context documented here. More broadly, our findings highlight the critical importance of comprehensive geographic sampling for interpreting discordant lines of evidence and evaluating species boundaries in recently diversified Amazonian primates.

## Data Availability

The occurrence records and associated data supporting the conclusions of this study are available from the corresponding author upon reasonable request.
